# Difficult Cholecystectomy for Acute Cholecystitis: Preoperative Risk Factors, Computed Tomography (CT) Findings, and Surgical Approach Selection in a Real-World Cohort

**DOI:** 10.7759/cureus.113634

**Published:** 2026-07-30

**Authors:** Motoyasu Tabuchi, Yurika Hata, Shuta Tamura, Sunao Uemura, Teppei Tokumaru, Rika Yoshimatsu, Takehiro Okabayashi

**Affiliations:** 1 Department of Gastroenterological Surgery, Kochi Health Sciences Center, Kochi, JPN; 2 Department of Gastrointestinal Surgery, Kochi Health Sciences Center, Kochi, JPN; 3 Department of Surgery, Kochi Health Sciences Center, Kochi, JPN; 4 Department of Radiology, Kochi Health Sciences Center, Kochi, JPN

**Keywords:** acute cholecystitis, cholecystectomy, computed tomography, c-reactive protein, difficult cholecystectomy

## Abstract

Background: Preoperative assessment of surgical difficulty is important in acute cholecystitis. Although laparoscopic cholecystectomy is the standard approach, real-world emergency surgery includes both laparoscopic and open cholecystectomy. This study evaluated preoperative factors associated with difficult cholecystectomy in a real-world surgical cohort.

Methods: This single-center retrospective study included 282 patients who underwent cholecystectomy for acute cholecystitis between January 2017 and December 2022. Difficult cholecystectomy was defined using an approach-specific composite score consisting of operative time, intraoperative blood loss, surgery-related postoperative complications, and subtotal cholecystectomy. Preoperative computed tomography (CT) findings were evaluated using five predefined findings: gallbladder wall thickening ≥5 mm, poor wall enhancement, pericholecystic fat stranding ≥5 mm, intraluminal or intramural gas, and pericholecystic abscess formation.

Results: Of the 282 patients, 79 (28.0%) were classified as having difficult cholecystectomy. In multivariable analysis, American Society of Anesthesiologists physical status ≥3 (odds ratio (OR), 2.25; 95% confidence interval (CI), 1.19-4.24; p = 0.012) and C-reactive protein level (OR, 1.04; 95% CI, 1.01-1.08; p = 0.015) were independently associated with difficult cholecystectomy. Positive CT findings showed a borderline association but did not reach statistical significance (OR, 1.86; 95% CI, 0.93-3.74; p = 0.082). Open cholecystectomy was more frequently selected for patients with severe clinical and inflammatory backgrounds, but severe complications and bile duct injury did not differ significantly between approaches.

Conclusions: In this real-world cohort, difficult cholecystectomy was mainly associated with systemic inflammatory burden and patient condition, whereas CT findings provided complementary information on local inflammation. Open cholecystectomy was selected for more severe cases without an increase in severe complications or bile duct injury, suggesting that surgical approach selection may be an important component of safe management in severe acute cholecystitis.

## Introduction

Acute cholecystitis is one of the most common abdominal emergency diseases encountered in daily clinical practice, and cholecystectomy remains the definitive treatment. Laparoscopic cholecystectomy is generally regarded the standard surgical approach because of its advantages, including reduced postoperative pain, shorter hospital stays, faster recovery, and fewer wound-related complications [1,2]. However, in patients with severe local inflammation, laparoscopic cholecystectomy can be technically challenging and may be associated with prolonged operative time, increased intraoperative blood loss, subtotal cholecystectomy, conversion to open surgery, and high risk of complications such as bile duct injury, bile leakage, and abdominal abscess.

Preoperative assessment of surgical difficulty in acute cholecystitis is clinically important. Accurate risk stratification may help surgeons plan operative strategies, assign experienced surgeons, prepare bailout procedures such as subtotal cholecystectomy, optimize operating room management, and provide appropriate preoperative counseling to patients and their families.

Previous studies and guidelines have mainly addressed difficult laparoscopic cholecystectomy, safe laparoscopic procedures, bailout strategies, risk factors for conversion to open surgery, and postoperative outcomes after laparoscopic cholecystectomy [3-5]. However, in real-world emergency surgery for acute cholecystitis, the choice between laparoscopic and open surgery is influenced not only by disease severity but also by surgeon availability, surgeon experience and preference, operating room constraints, anesthetic considerations, and institutional treatment policies. Therefore, studies limited to laparoscopic cholecystectomy alone may not fully reflect emergency surgical practice in regional referral or general hospitals.

Several factors have been reported to be associated with difficult cholecystectomy, including age, sex, body mass index, American Society of Anesthesiologists physical status (ASA-PS), white blood cell count, C-reactive protein (CRP) level, symptom duration, and Tokyo Guidelines severity grade [4-8]. Imaging findings are also important because surgical difficulty is largely influenced by local inflammation and anatomical changes. Previous studies have reported that computed tomography (CT) findings such as gallbladder wall thickening, poor wall enhancement, pericholecystic fluid collection or abscess formation, emphysematous changes, and pericholecystic inflammatory changes are associated with inflammatory severity and surgical difficulty [3,8-11].

However, not all CT findings have the same clinical significance. Mild inflammatory findings, such as gallbladder wall thickening and pericholecystic fat stranding, may indicate acute inflammation, whereas poor wall enhancement, intraluminal or intramural gas, and pericholecystic abscess formation may reflect gangrenous or complicated cholecystitis. Therefore, distinguishing severe CT findings from nonspecific inflammatory findings may be useful for the preoperative assessment of a difficult surgical course.

The primary objective of this study was to identify preoperative clinical and CT-based factors associated with a difficult surgical course in patients undergoing cholecystectomy for acute cholecystitis. The secondary objectives were to evaluate the association between CT grade and difficult surgical course and to compare the clinical characteristics and perioperative outcomes between laparoscopic and open cholecystectomy. To reflect real-world emergency surgical practice, both laparoscopic and open cholecystectomy cases were included. In this study, difficulty was assessed using a newly developed approach-specific composite score that included operative time, intraoperative blood loss, surgery-related postoperative complications, and subtotal cholecystectomy. Therefore, the outcome should be interpreted as a difficult surgical course rather than pure intraoperative technical difficulty.

## Materials and methods

Study design and patients

This was a single-center retrospective observational study including 282 patients who underwent cholecystectomy for acute cholecystitis between January 2017 and December 2022. Clinical records, operative reports, laboratory data, and preoperative imaging findings were retrospectively reviewed.

The collected variables included age, sex, body mass index, ASA-PS classification [12], antithrombotic medication use, history of previous abdominal surgery, age-adjusted Charlson comorbidity index (CCI) [13,14], white blood cell count, CRP level, performance of percutaneous transhepatic gallbladder drainage, Tokyo Guidelines 2018 (TG18) severity grade [8], symptom duration >72 hours, preoperative CT findings, primary operator status, surgical approach, operative time, intraoperative blood loss, subtotal cholecystectomy, postoperative complications graded according to the Clavien-Dindo classification [15], and postoperative length of hospital stay.

To reflect real-world emergency surgical practice, both laparoscopic and open cholecystectomy cases were included in this study. At our institution, the initial surgical approach was selected by the attending surgical team based on the patient’s general condition, severity of inflammation, preoperative imaging findings, expected technical difficulty, surgeon experience, operating room availability, anesthetic considerations, and institutional practice patterns. Primary open cholecystectomy was considered for selected patients with severe local inflammation, suspected difficult anatomy, poor physiological reserve, or other clinical factors that made a laparoscopic approach less suitable. Therefore, surgical approach was considered a treatment-related variable rather than a pure preoperative predictor and was not included in the primary multivariable model. For the comparison between laparoscopic and open cholecystectomy, surgical approach was classified according to the initial operative approach.

CT findings and CT grade

**Figure 1:**
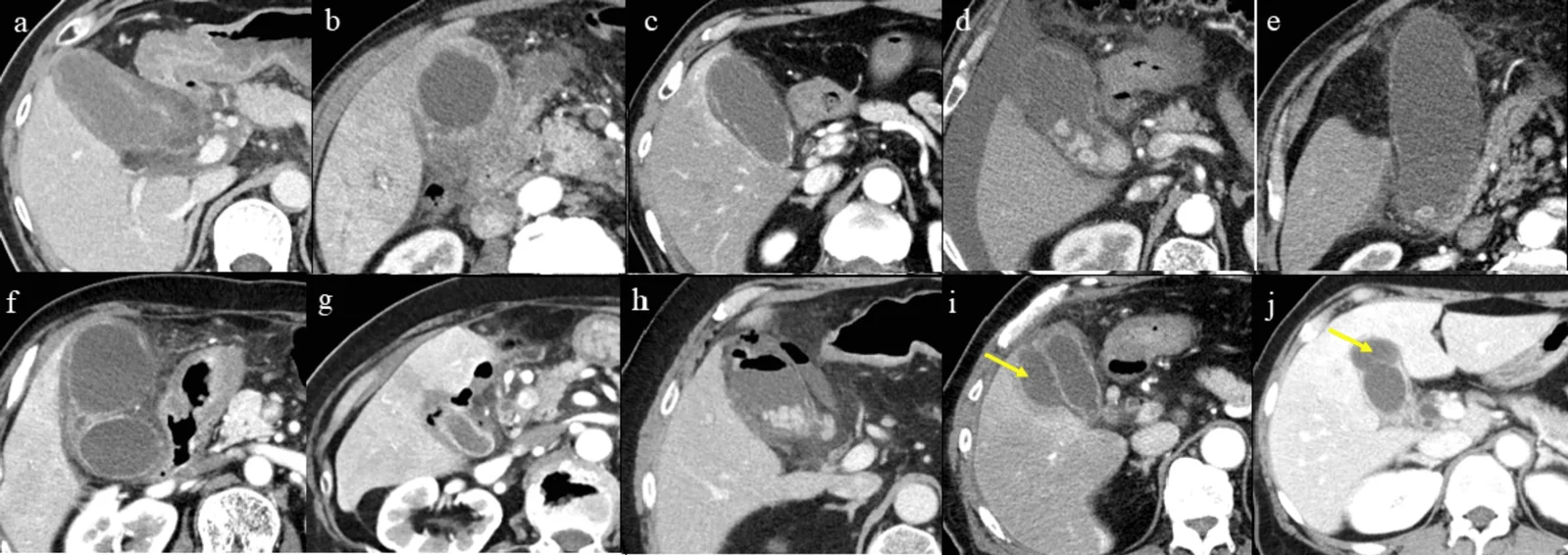
Representative preoperative CT findings used to assess local inflammatory severity in acute cholecystitis (a,b) Gallbladder wall thickening ≥5 mm. The thickened gallbladder wall is shown in two representative CT images. (c,d) Poor or absent gallbladder wall enhancement on contrast-enhanced CT, suggesting ischemic or gangrenous change. (e,f) Pericholecystic fat stranding extending ≥5 mm from the gallbladder wall, indicating surrounding inflammatory change. (g,h) Intraluminal or intramural gas within the gallbladder. (i,j) Pericholecystic abscess formation. Arrows indicate the abscess cavity CT, computed tomography

We developed a CT grading system based on preoperative CT findings. The evaluated CT findings included five components: gallbladder wall thickening ≥5 mm, poor gallbladder wall enhancement, pericholecystic fat stranding extending ≥5 mm from the gallbladder wall, intraluminal or intramural gas, and pericholecystic abscess formation (Figure 1). Each finding was assigned 1 point, and the sum of these five findings was defined as the CT grade. The presence of at least one of these five findings was defined as positive CT findings. Gallbladder neck stone impaction was recorded separately and was not included in the CT grade, as it was considered an obstructive finding rather than an inflammatory finding.

Preoperative CT images obtained before cholecystectomy were used for the imaging assessment. When multiple CT examinations were available, the CT examination closest to surgery was used. Contrast-enhanced findings, including poor gallbladder wall enhancement, were assessed when contrast-enhanced CT images were available.

CT images were independently reviewed by two surgeons and one radiologist who were blinded to the clinical and operative outcomes. The reviewers assessed only the imaging findings. Discrepancies in CT interpretation were resolved by consensus, and the final CT findings were used for analysis.

Definition of difficult surgical course

Surgical difficulty was assessed using an approach-specific difficult surgical course score consisting of four components: operative time, intraoperative blood loss, surgery-related postoperative complications, and subtotal cholecystectomy.

Because the distributions of operative time and intraoperative blood loss differed substantially between laparoscopic and open cholecystectomy, these variables were scored using surgical approach-specific percentile thresholds. Values below the 75th percentile were assigned 0 points, values between the 75th and 89th percentiles were assigned 2 points, and values at or above the 90th percentile were assigned 4 points.

For laparoscopic cases, the 75th and 90th percentile thresholds were 154 and 192.4 minutes for operative time and 130 and 300 mL for intraoperative blood loss, respectively. For open cases, the corresponding thresholds were 108 and 133 minutes for operative time and 325 and 526 mL for intraoperative blood loss, respectively.

Surgery-related postoperative complications included bile duct injury, postoperative bile leakage, postoperative bleeding, intra-abdominal abscess, and surgical site infection. These complications were scored according to the Clavien-Dindo classification [15] as follows: no complication, 0 points; Clavien-Dindo grade I-II, 2 points; and Clavien-Dindo grade ≥IIIa, 4 points. Cases in which subtotal cholecystectomy was ultimately performed were assigned 4 points.

Subtotal cholecystectomy was determined based on operative records and photographs of the resected gallbladder specimen. For laparoscopic cholecystectomy, intraoperative videos were also reviewed. All cases requiring subtotal cholecystectomy were assigned 4 points.

Patients with a total score of ≥4 were classified as having a difficult surgical course, whereas those with a total score of <4 were classified as having a nondifficult surgical course (Table 1). The cutoff value of ≥4 points was selected because it identifies patients with at least one major difficult component, such as operative time or intraoperative blood loss at or above the 90th percentile, surgery-related postoperative complications of Clavien-Dindo grade ≥IIIa, or subtotal cholecystectomy, as well as patients with a combination of moderate difficult components. This score was developed to capture an overall difficult surgical course rather than pure intraoperative technical difficulty.

**Table 1:** Definition of the difficult cholecystectomy score Values below the 75th percentile were assigned 0 points, values from the 75th-89th percentile were assigned 2 points, and values at or above the 90th percentile were assigned 4 points for operative time and intraoperative blood loss. Patients with a total score ≥4 were classified as having difficult cholecystectomy DC, difficult cholecystectomy

Component	Criteria	Points
Operative time		
Laparoscopic	<154/154-192.3/≥192.4 minutes	0/2/4
Open	<108/108-132.9/≥133 minutes	0/2/4
Intraoperative blood loss		
Laparoscopic	<130/130-299 mL/≥300 mL	0/2/4
Open	<325/325-525/≥526 mL	0/2/4
Surgery-related postoperative complication	None/Clavien-Dindo grade I-II/Clavien-Dindo grade ≥IIIa	0/2/4
Subtotal cholecystectomy	Not performed/performed	0/4
Definition of difficult cholecystectomy	Total score <4/≥4	Non-DC/DC

Statistical analysis

Continuous variables are presented as medians with interquartile ranges (IQRs), and categorical variables are presented as numbers and percentages. Comparisons between the difficult and nondifficult surgical course groups were performed using the Mann-Whitney U test for continuous variables and the chi-square test or Fisher’s exact test for categorical variables, as appropriate.

Multivariable logistic regression analysis was performed to identify preoperative factors associated with a difficult surgical course. TG18 severity grade itself was not included in the model because it partly incorporates CT-based local inflammatory findings, which overlapped with the CT findings evaluated in this study. Instead, clinically relevant preoperative variables and TG18-related components were entered into the model, including ASA-PS ≥3, age-adjusted CCI, CRP level, elevated WBC count ≥18,000/mm³, organ failure corresponding to TG18 grade III, symptom duration >72 hours, and positive CT findings.

As sensitivity analyses, the multivariable logistic regression analysis was repeated using a stricter cutoff value of ≥6 points for the composite difficulty score and using a modified composite score that excluded postoperative complications. The same explanatory variables as those in the primary multivariable model were used. All statistical analyses were performed using Easy R (R Foundation for Statistical Computing, Vienna, Austria) [16]. All tests were two-sided, and a p value <0.05 was considered statistically significant.

Ethics statement

This study was approved by the Institutional Review Board of Kochi Health Sciences Center (approval no. 261021) and was conducted in accordance with the ethical standards of the Declaration of Helsinki. The requirement for written informed consent was waived because of the retrospective nature of the study. Information regarding the study was disclosed on the institutional website, and patients were given the opportunity to opt out.

## Results

Among the 282 patients included in this study, 79 patients were classified as having a difficult surgical course according to the approach-specific composite score, and 203 patients were classified as having a nondifficult surgical course.

Patients with a difficult surgical course had a higher proportion of ASA-PS ≥3 than those without a difficult surgical course (37/79, 46.8% vs. 53/203, 26.1%; p = 0.001). CRP levels were also higher in the difficult surgical course group (17.3 mg/dL; IQR, 10.8-23.3 vs. 9.1 mg/dL, IQR, 1.5-18.1; p < 0.001). Symptom duration >72 hours was more frequent in the difficult surgical course group (29/79, 36.7% vs. 45/203, 22.2%; p = 0.019), as were positive CT findings (63/79, 79.7% vs. 114/203, 56.2%; p < 0.001) (Table 2). The rate of difficult surgical course was similar between laparoscopic and open cases after applying approach-specific thresholds for operative time and blood loss: 46 of 169 laparoscopic cases (27.2%) and 33 of 113 open cases (29.2%) were classified as having a difficult surgical course.

**Table 2:** Patient characteristics according to difficult cholecystectomy Data are presented as median (interquartile range) for continuous variables and n (%) for categorical variables. p values were calculated using the Mann-Whitney U test for continuous variables and the chi-square test or Fisher’s exact test for categorical variables, as appropriate. Chi-square values are reported when the chi-square test was used. p values <0.05 were considered statistically significant ASA-PS, American Society of Anesthesiologists physical status; BMI, body mass index; CCI, Charlson comorbidity index; CD, Clavien-Dindo; CRP, C-reactive protein; CT, computed tomography; DC, difficult cholecystectomy; PTGBD, percutaneous transhepatic gallbladder drainage; TG18, Tokyo Guidelines 2018; WBC, white blood cell

Variable	Non-DC (n = 203)	DC (n = 79)	Test statistic	p value
Age, years	73 (64-82.5)	75 (68.5-82)	U = 7393.0	0.309
Male sex	133 (65.5)	55 (69.6)	χ² = 0.27	0.606
BMI, kg/m²	24.5 (22.3-27.9)	24 (21.6-27.3)	U = 7946.0	0.809
ASA-PS ≥3	53 (26.1)	37 (46.8)	χ² = 10.31	0.001
Antithrombotic medication	69 (34.0)	27 (34.2)	χ² = 0.00	1
Previous abdominal surgery	56 (27.6)	27 (34.2)	χ² = 0.89	0.345
Age-adjusted CCI	4 (3-5)	4 (3-5)	U = 7757.0	0.665
WBC count/mm³	12,890 (10,065-16,775)	13,700 (10,030-17,530)	U = 7685.5	0.589
WBC >18,000/mm³	40 (19.7)	19 (24.1)	χ² = 0.41	0.52
CRP, mg/dL	9.1 (1.5-18.1)	17.3 (10.8-23.3)	U = 5053.5	<0.001
PTGBD	2 (1.0)	3 (3.8)	Fisher’s exact test	0.136
TG18				
Grade I	107 (52.7)	24 (30.4)	χ² = 11.41, df = 2	0.003
Grade II	86 (42.4)	49 (62.0)		
Grade III	10 (4.9)	6 (7.6)		
Symptom duration >72 hours	45 (22.2)	29 (36.7)	χ² = 5.48	0.019
CT finding positive	114 (56.2)	63 (79.7)	χ² = 12.55	<0.001
Gallbladder neck stone impaction	41 (20.2)	16 (20.3)	χ² = 0.00	1
Organ failure (Grade III)	10 (4.9)	6 (7.6)	Fisher’s exact test	0.397
Primary operator: consultant	112 (55.2)	39 (49.4)	χ² = 0.55	0.456
Laparoscopic approach	123 (60.6)	46 (58.2)	χ² = 0.05	0.819
Operative time, minute	97 (77-121.5)	141 (111-180)	U = 3403.5	<0.001
Intraoperative blood loss, mL	50 (0-130)	260 (145-492.5)	U = 2942.5	<0.001
Subtotal cholecystectomy	0 (0.0)	19 (24.1)	Fisher’s exact test	<0.001
Postoperative complication, any	19 (9.4)	23 (29.1)	χ² = 15.98	<0.001
Severe complication (CD ≥IIIa)	8 (3.9)	12 (15.2)	χ² = 9.28	0.002
Postoperative hospital stay, days	6 (5-9)	9 (6-14.5)	U = 5126.5	<0.001

Multivariable analysis revealed that ASA-PS ≥3 (odds ratio (OR), 2.25; 95% confidence interval (CI), 1.19-4.24; p = 0.012) and CRP (OR, 1.04; 95% CI, 1.01-1.08; p = 0.015) were independently associated with a difficult surgical course. In contrast, age-adjusted CCI (OR, 0.92; 95% CI, 0.77-1.11; p = 0.380), WBC ≥18,000/mm³ (OR, 0.98; 95% CI, 0.48-2.00; p = 0.960), organ failure corresponding to TG18 grade III (OR, 0.83; 95% CI, 0.25-2.70; p = 0.760), symptom duration >72 hours (OR, 1.69; 95% CI, 0.91-3.13; p = 0.096), and positive CT findings (OR, 1.86; 95% CI, 0.93-3.74; p = 0.082) were not independently associated with a difficult surgical course (Table 3).

**Table 3:** Multivariable logistic regression analysis for difficult cholecystectomy Odds ratios are presented with 95% CIs. Wald z-statistics are reported for each variable. Odds ratios for CRP and age-adjusted CCI are presented per 1-unit increase. TG18 severity grade itself was not included in the model because it partly incorporates CT-based local inflammatory findings, which overlapped with the CT findings evaluated in this study. Positive CT findings were defined as the presence of at least one of the five predefined CT findings. p values <0.05 were considered statistically significant ASA-PS, American Society of Anesthesiologists physical status; CCI, Charlson comorbidity index; CI, confidence interval; CRP, C-reactive protein; CT, computed tomography; TG18, Tokyo Guidelines 2018; WBC, white blood cell

Factor	Odds ratio	95% CI	Wald z	p value
ASA-PS ≥3	2.25	1.19-4.24	2.5	0.012
Age-adjusted CCI	0.92	0.77-1.11	-0.89	0.38
CRP, mg/dL	1.04	1.01-1.08	2.29	0.015
Elevated WBC count ≥18,000/mm^3^	0.98	0.48-2.00	-0.06	0.96
Organ failure, Grade III	0.83	0.25-2.70	-0.31	0.76
Symptom duration >72 hours	1.69	0.91-3.13	1.67	0.096
CT finding positive	1.86	0.93-3.74	1.75	0.082

Sensitivity analyses are shown in Tables 4, 5. In the sensitivity analysis using a stricter cutoff value of ≥6 points for the composite difficulty score, CRP remained significantly associated with a difficult surgical course (OR, 1.05; 95% CI, 1.00-1.10; p = 0.032), while positive CT findings showed a borderline association but did not reach statistical significance (OR, 2.89; 95% CI, 1.00-8.38; p = 0.051). In the sensitivity analysis using a modified composite score excluding postoperative complications, positive CT findings (OR, 2.73; 95% CI, 1.39-5.35; p = 0.0034) and symptom duration >72 hours (OR, 2.04; 95% CI, 1.09-3.82; p = 0.027) were independently associated with difficulty.

**Table 4:** Sensitivity analysis using a modified composite score excluding postoperative complications Odds ratios are presented with 95% CIs. Wald z-statistics are reported for each variable. Odds ratios for CRP and age-adjusted CCI are presented per one-unit increase. In this sensitivity analysis, postoperative complications were excluded from the composite difficulty score. The same explanatory variables as those in the primary multivariable model were used. p values <0.05 were considered statistically significant ASA-PS, American Society of Anesthesiologists physical status; CCI, Charlson comorbidity index; CI, confidence interval; CRP, C-reactive protein; CT, computed tomography; TG18, Tokyo Guidelines 2018; WBC, white blood cell

Factor	Odds ratio	95% CI	Wald z	p value
ASA-PS ≥3	1.49	0.77-2.87	1.19	0.24
Age-adjusted CCI	1.07	0.88-1.30	0.68	0.49
CRP, mg/dL	1.03	1.00-1.07	1.71	0.056
Elevated WBC count ≥18,000/mm³	0.61	0.28-1.32	-1.25	0.21
Organ failure, TG18 grade III	1.41	0.44-4.56	0.58	0.57
Symptom duration >72 hours	2.04	1.09-3.82	2.23	0.028
CT finding positive	2.73	1.39-5.35	2.92	0.0034

**Table 5:** Sensitivity analysis using a stricter cutoff value of ≥6 points for the composite difficulty score Odds ratios are presented with 95% CIs. Wald z-statistics are reported for each variable. Odds ratios for CRP and age-adjusted CCI are presented per one-unit increase. In this sensitivity analysis, a difficult surgical course was defined as a composite difficulty score ≥6 points. The same explanatory variables as those in the primary multivariable model were used. p values <0.05 were considered statistically significant ASA-PS, American Society of Anesthesiologists physical status; CCI, Charlson comorbidity index; CI, confidence interval; CRP, C-reactive protein; CT, computed tomography; TG18, Tokyo Guidelines 2018; WBC, white blood cell

Factor	Odds ratio	95% CI	Wald z	p value
ASA-PS ≥3	1.83	0.75-4.46	1.33	0.18
Age-adjusted CCI	0.96	0.72-1.29	-0.27	0.8
CRP, mg/dL	1.05	1.00-1.10	2.01	0.032
Elevated WBC count ≥18,000/mm³	1.33	0.51-3.46	0.58	0.56
Organ failure, TG18 Grade III	1.22	0.28-5.29	0.27	0.8
Symptom duration >72 hours	1.23	0.51-3.00	0.46	0.64
CT finding positive	2.89	1.00-8.38	1.96	0.051

The relationship between CT grade and difficult surgical course is shown in Table 6. The rate of difficult surgical course was 15.2% (16/105) in patients with CT grade 0, 26.2% (17/65) in grade 1, 40.0% (28/70) in grade 2, 45.5% (15/33) in grade 3, 25.0% (2/8) in grade 4, and 100.0% (1/1) in grade 5. Overall, the distribution of difficult surgical course differed significantly across CT grades (p < 0.001). Although the number of patients with CT grade 4 or 5 was limited, the rate of difficult surgical course increased from CT grade 0 to grade 3.

**Table 6:** Difficult cholecystectomy rate according to CT grade score Data are presented as n and %. CT grade was defined as the sum of five predefined CT findings: gallbladder wall thickening ≥5 mm, poor gallbladder wall enhancement, pericholecystic fat stranding ≥5 mm, intraluminal or intramural gas, and pericholecystic abscess formation. Each finding was assigned 1 point. The chi-square test was used to compare the distribution of difficult cholecystectomy across CT grades. p values <0.05 were considered statistically significant CT, computed tomography; DC, difficult cholecystectomy

CT grade score	Total (n)	DC (n)	DC rate (%)
0	105	16	15.2
1	65	17	26.2
2	70	28	40
3	33	15	45.5
4	8	2	25
5	1	1	100
Overall comparison	-	χ² = 21.18, df = 5	p < 0.001

Individual CT findings associated with difficult surgical course are shown in Table 7. Any positive CT finding was more frequent in the difficult surgical course group than in the nondifficult surgical course group (63/79, 79.7% vs. 114/203, 56.2%; p < 0.001). Among individual CT findings, poor or absent gallbladder wall enhancement was significantly more frequent in the difficult surgical course group (33/79, 41.8% vs. 54/203, 26.6%; p = 0.020), as were pericholecystic fat stranding ≥5 mm (48/79, 60.8% vs. 76/203, 37.4%; p < 0.001) and pericholecystic abscess formation (14/79, 17.7% vs. 14/203, 6.9%; p = 0.012). In contrast, gallbladder wall thickening ≥5 mm (29/79, 36.7% vs. 55/203, 27.1%; p = 0.150), intraluminal or intramural gas (6/79, 7.6% vs. 8/203, 3.9%; p = 0.227), and gallbladder neck stone impaction (16/79, 20.3% vs. 41/203, 20.2%; p = 1.000) were not significantly associated with difficult surgical course.

**Table 7:** Individual CT findings according to difficult cholecystectomy Data are presented as n (%). Positive CT findings were defined as the presence of at least one of the five predefined CT findings. p values were calculated using the chi-square test or Fisher’s exact test, as appropriate. Chi-square values are reported when the chi-square test was used. p values <0.05 were considered statistically significant CT, computed tomography; DC, difficult cholecystectomy; GB, gallbladder

CT finding	Positive n	DC among positive, n (%)	Non-DC positive, n (%)	Test statistic	p value
Any CT finding	177	63 (79.7)	114 (56.2)	χ² = 12.55	<0.001
Wall thickening ≥5 mm	84	29 (36.7)	55 (27.1)	χ² = 2.08	0.15
Poor/nonenhancement of GB wall	87	33 (41.8)	54 (26.6)	χ² = 5.45	0.02
Pericholecystic fat stranding ≥5 mm	124	48 (60.8)	76 (37.4)	χ² = 11.63	<0.001
Intraluminal or intramural gas	14	6 (7.6)	8 (3.9)	Fisher’s exact test	0.227
Pericholecystic abscess	28	14 (17.7)	14 (6.9)	χ² = 6.29	0.012
GB neck stone impaction	57	16 (20.3)	41 (20.2)	Fisher’s exact test	1

The comparison between laparoscopic and open cholecystectomy is shown in Table 8. Open cholecystectomy was selected for patients with more severe clinical and inflammatory backgrounds. Compared with the laparoscopic cholecystectomy group, the open cholecystectomy group was older (78 years, IQR, 70-84 vs. 71 years, IQR, 61-80; p < 0.001) and had higher proportions of ASA-PS ≥3 (51/113, 45.1% vs. 39/169, 23.1%; p < 0.001), symptom duration >72 hours (40/113, 35.4% vs. 34/169, 20.1%; p = 0.007), and positive CT findings (86/113, 76.1% vs. 91/169, 53.8%; p < 0.001). The open cholecystectomy group also had higher age-adjusted CCI and CRP levels than the laparoscopic cholecystectomy group.

**Table 8:** Comparison between laparoscopic and open cholecystectomy Data are presented as median (interquartile range) for continuous variables and n (%) for categorical variables. Surgical approach was classified according to the initial operative approach. p values were calculated using the Mann-Whitney U test for continuous variables and the chi-square test or Fisher’s exact test for categorical variables, as appropriate. Chi-square values are reported for categorical variables when the chi-square test was used. p values <0.05 were considered statistically significant ASA-PS, American Society of Anesthesiologists physical status; CCI, Charlson comorbidity index; CD, Clavien-Dindo; CRP, C-reactive protein; CT, computed tomography

Variable	Laparoscopic (n = 169)	Open (n = 113)	Test statistic	p value
Age, years	71 (61-80)	78 (70-84)	U = 6642.0	<0.001
Male sex	113 (66.9)	75 (66.4)	Fisher’s exact test	1
ASA-PS ≥3	39 (23.1)	51 (45.1)	χ² = 14.16	<0.001
Age-adjusted CCI	3 (2-4)	4 (3-5)	U = 6844.0	<0.001
CRP, mg/dL	9.2 (1.3-18.2)	16.2 (6.6-21.0)	U = 7016.5	<0.001
Symptom duration >72 hours	34 (20.1)	40 (35.4)	χ² = 7.40	0.007
CT finding positive	91 (53.8)	86 (76.1)	χ² = 13.42	<0.001
Gallbladder neck stone impaction	39 (23.1)	18 (15.9)	χ² = 1.73	0.189
Difficult cholecystectomy	46 (27.2)	33 (29.2)	χ² = 0.05	0.819
Operative time, min	121 (96-154)	85 (68-108)	U = 14265.5	<0.001
Intraoperative blood loss, mL	30 (0-130)	180 (55-325)	U = 5149.5	<0.001
Subtotal cholecystectomy	8 (4.7)	11 (9.7)	χ² = 1.96	0.162
Bile duct injury	2 (1.2)	0 (0.0)	Fisher’s exact test	0.518
Postoperative complication, any	18 (10.7)	24 (21.2)	χ² = 5.18	0.023
Postoperative hospital stay, days	6 (4-9)	9 (7-12)	U = 5325.5	<0.001
Severe postoperative complication (CD ≥ IIIa)	11 (6.5)	9 (8.0)	Fisher’s exact test	0.644

Operative time was shorter in the open cholecystectomy group, whereas intraoperative blood loss and postoperative hospital stay were greater than in the laparoscopic cholecystectomy group. The rate of difficult surgical course did not differ significantly between the two groups after applying approach-specific thresholds for operative time and intraoperative blood loss (33/113, 29.2% vs. 46/169, 27.2%; p = 0.819). Postoperative complications of any grade were more frequent in the open cholecystectomy group (24/113, 21.2% vs. 18/169, 10.7%; p = 0.023). However, the rate of severe postoperative complications did not differ significantly between open and laparoscopic cholecystectomy (9/113, 8.0% vs. 11/169, 6.5%; p = 0.644). Bile duct injury occurred in two patients in the laparoscopic cholecystectomy group and in none of the patients in the open cholecystectomy group (2/169, 1.2% vs. 0/113, 0.0%; p = 0.518).

## Discussion

This study evaluated difficult surgical course in a real-world cohort that included both laparoscopic and open cholecystectomy for acute cholecystitis. The main findings were as follows: first, ASA-PS ≥3 and CRP level were independently associated with a difficult surgical course; second, CT findings were more frequent in difficult cases but were not independent predictors after adjustment for clinical factors; and third, open cholecystectomy was more frequently selected for patients with more severe clinical and inflammatory backgrounds, without an increase in severe postoperative complications or bile duct injury. These findings suggest that difficulty in cholecystectomy should be evaluated not only as a prediction problem but also as part of real-world surgical decision-making.

CRP reflects the inflammatory burden, including both local and systemic inflammation, whereas ASA-PS represents the patient’s physiological reserve, comorbidity burden, and tolerance to emergency surgery. The association of elevated CRP levels and poor ASA-PS with a difficult surgical course is consistent with previous reports [5,11]. These findings suggest that surgical difficulty in acute cholecystitis is determined not only by the severity of local inflammation but also by patient background and systemic condition. The sensitivity analyses provided additional insight into the interpretation of the composite difficulty score. In the stricter cutoff model using ≥6 points, CRP remained significantly associated with a difficult surgical course, supporting the role of systemic inflammatory burden. In contrast, when postoperative complications were excluded from the composite score, positive CT findings and symptom duration >72 hours became independently associated with difficulty. These findings suggest that local inflammatory factors may be particularly important when difficulty is defined more closely to intraoperative factors, whereas the primary composite outcome reflects an overall difficult surgical course that includes postoperative outcomes.

Although CT findings did not remain independent predictors after adjustment for clinical factors, the rate of difficult surgical course tended to increase with higher CT grades, particularly from CT grade 0-3. Among individual CT findings, poor gallbladder wall enhancement, pericholecystic fat stranding, and pericholecystic abscess formation were more frequently observed in the difficult surgical course group than in the nondifficult surgical course group. These findings suggest that CT may still be useful for assessing the extent of local inflammation and anticipating surgical difficulty. However, because the number of patients with CT grades 4 and 5 was small, the present results should not be interpreted as demonstrating a consistent dose-response relationship across all CT grades.

Previous studies have evaluated the usefulness of preoperative CT findings for predicting difficult laparoscopic cholecystectomy and conversion to open surgery. Fuks et al. reported that poor gallbladder wall enhancement and gallstone impaction in the infundibulum were associated with conversion from laparoscopic to open cholecystectomy [17]. Maehira et al. showed that increased arterial-phase enhancement of the liver adjacent to the gallbladder on dynamic CT predicted difficult laparoscopic cholecystectomy in acute cholecystitis [18]. More recently, radiomics-based models and radiological nomograms have also been developed to predict difficult laparoscopic cholecystectomy [19,20]. In contrast, Chung et al. recently reported that CT findings alone did not independently predict surgical difficulty, whereas clinical factors such as CRP were more strongly associated with difficult laparoscopic cholecystectomy [21]. Our findings are consistent with this observation and support the concept that CT findings should be interpreted as complementary information in combination with clinical and laboratory findings.

In routine clinical practice, the choice between laparoscopic and open cholecystectomy may be influenced by disease severity as well as practical factors such as surgeon experience, operating room availability, anesthetic considerations, and institutional practice patterns [1,3,22]. Therefore, the present study should be interpreted as an evaluation of the difficult surgical course in real-world acute cholecystitis surgery, rather than as an assessment of pure technical difficulty in laparoscopic cholecystectomy alone.

Although the open cholecystectomy group in our cohort included patients with more severe clinical and inflammatory backgrounds, the rates of severe postoperative complications and bile duct injury did not differ significantly compared with the laparoscopic cholecystectomy group. This finding suggests that open cholecystectomy may remain an important safety-oriented option for selected patients with severe inflammation or unfavorable clinical conditions.

Previous randomized trials and meta-analyses comparing laparoscopic and open cholecystectomy have shown that laparoscopic cholecystectomy is safe and offers advantages in terms of shorter hospital stay and faster recovery [2,23,24]. Therefore, laparoscopic cholecystectomy remains the first-line surgical approach for acute cholecystitis. However, in selected patients with severe inflammation, complex anatomy, or unfavorable clinical conditions, open cholecystectomy may still have an important role as a safety-oriented surgical option [25].

This study has several limitations. First, this was a single-center retrospective study, and selection bias could not be eliminated. Second, the choice of surgical approach was not standardized and was influenced by surgeon- and institution-related factors. Although this reflects real-world emergency surgical practice, it limits causal interpretation regarding the safety or superiority of laparoscopic or open cholecystectomy. Third, the composite difficulty score used in this study was newly developed and has not been externally validated. Because surgery-related postoperative complications were included in the score, the primary outcome reflects a difficult surgical course rather than pure intraoperative technical difficulty. Although sensitivity analyses using a stricter cutoff value and a modified score excluding postoperative complications were performed, further external validation is required before this score can be widely applied. Fourth, CT findings were reviewed by two surgeons and one radiologist and finalized by consensus, but interobserver agreement was not quantitatively assessed because the individual preconsensus assessments were not available. This may limit the reproducibility of the imaging assessment. Fifth, the number of patients with high CT grades and the number of bile duct injury events were limited, and subgroup analyses according to surgical approach may have been underpowered. Further multicenter studies are needed to validate these findings.

## Conclusions

In conclusion, ASA-PS ≥3 and CRP level were the main preoperative factors associated with difficult cholecystectomy in patients with acute cholecystitis. CT findings were not independent predictors after adjustment for clinical factors, but they may provide supportive information regarding local inflammation. Open cholecystectomy may remain a safety-oriented option for selected patients with severe inflammation or unfavorable clinical conditions. These findings support a pragmatic strategy in which preoperative clinical severity, inflammatory burden, CT findings, and available surgical expertise are integrated when selecting the operative approach for acute cholecystitis.
